# Correction: Recency and rarity effects in disambiguating the focus of utterance: A developmental study

**DOI:** 10.1371/journal.pone.0324557

**Published:** 2025-05-16

**Authors:** The PLOS One Staff

Fig 1 is uploaded incorrectly. Please see the corrected version here. The publisher apologizes for the error.

**Fig 1:**
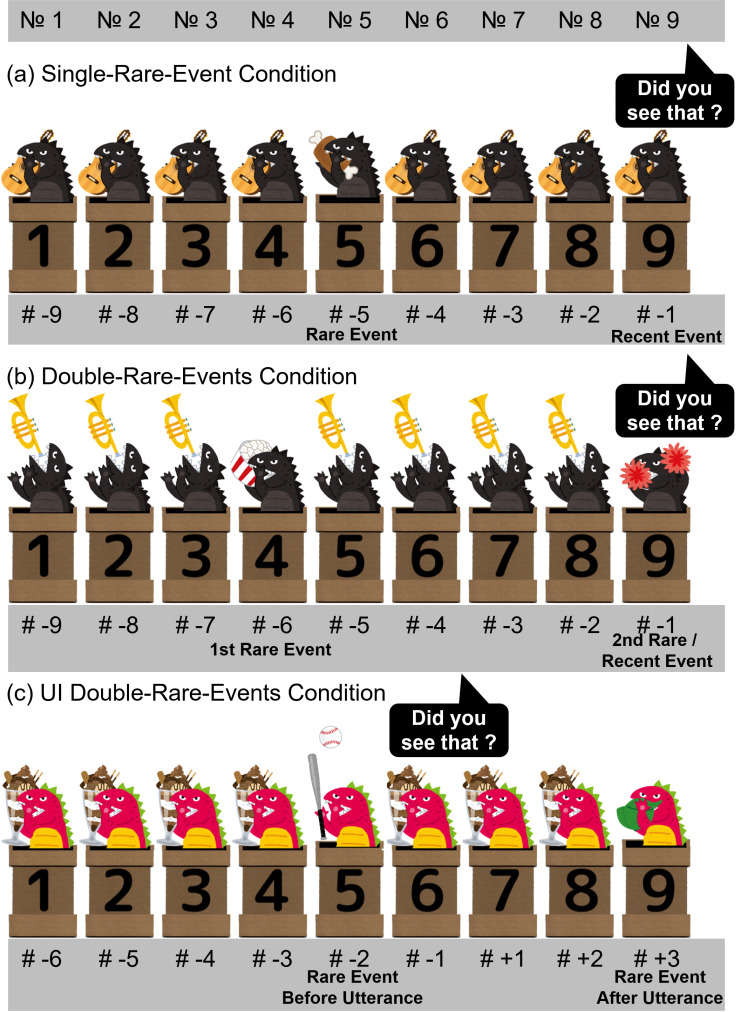
An example of the sequence of Single-Rare-Event Condition (a), Double Rare Events Condition (b) and UI Double Rare Events Condition (c), respectively. The numbers at the bottom denote the timing relative to the utterance. A negative value indicates a timing before the utterance, while a positive value signifies a timing after the utterance. Two kinds of monsters, black and red, were used alternately across trials so that participants would not lose interest. (a) An example of rare-event timing #-5. A monster emerged from a pipe, completed a specific action in 0.86s, and then disappeared back into the pipe. The next monster emerged after a 1-s interval. (b) An example of the rare-event timing #-6/ #-1. Two distinct actions were used for 1st rare event and 2nd rare event, respectively. (c) An example of the rare-event timing #-2/ # + 3.
