# Supplementary material for: Recency and rarity effects in disambiguating the focus of utterance: A developmental study
Source: PLoS One. 2025 Feb 12;20(2):e0317433. doi: 10.1371/journal.pone.0317433 (PMC11819549; doi:10.1371/journal.pone.0317433)
Supplement: S1 File — (PDF) [file pone.0317433.s001.pdf]

1. Practice Session Procedure

To be habituated to the experimental procedures, participants first completed a practice session. Participants were presented with the following videos (Figure S1). Four animals (cats and dogs) emerged from the pipe, paused for 0.86 s and disappeared sequentially from pipes #1 to #4. The events happened from left to right so that participants could easily process them. People in Japanese culture generally read an increasing list of numbers from left to right, and thus expect small numbers to appear on the left and large numbers to appear on the right (de Hevia et al., 2017; Di Giorgio et al., 2019). Immediately after the last appearance, a recorded voice “Did you see cat(s)?” was played.

The Japanese language has linguistic markers to denote the plural form, similar to the "s" in English. The markers should not necessarily be used for the plural form but rather when the distinction between singular or plural form is a critical aspect of the context. When words do not accompany such a marker, they can be singular or plural depending on the context (Sarnecka et al., 2007). The term "cat" carries ambiguity as to whether it refers to the singular or plural form. Thus, the referent of "cat " could potentially mean only one, a few, or all cats.

After the animation was presented on the monitor, the monitor became a blank white screen and the image, in which the four animals emerged, was presented on the touch screen (15.6 inch, Spkulia). Participants were asked to draw a yellow rectangle(s) around the character(s) they thought the initiator was referring to (reference

assignment task). The yellow rectangle was drawn by touching the screen and dragging the finger into a rectangular outline (Visual Basic 2010). Upon making a decision, participants had to press a decision button on the upper left side of the screen and then they proceeded to the memory task.

In the memory task, the monitor on the touch panel presented an identical image of the reference assignment task except that two red arrows were overlaid between #2 and #3, and next to #4. Participants marked the arrow which indicated the position of utterance by same procedure as the reference assignment task.

As the purpose of this phase was learning the task, the experimenter told participants how to manipulate the touch screen only when they needed help. At this time, the experimenter was careful not to guide their responses and only provided help when asked. Participants completed ten trials (The sequence of each trial is shown in Table S1).

References

de Hevia, M. D., Veggiotti, L., Streri, A., & Bonn, C. D. (2017). At birth, humans associate “few” with left and “many” with right. *Current Biology*, 27, 3879-3884.

Di Giorgio, E., Lunghi, M., Rugani, R., Regolin, L., Dalla Barba, B., Vallortigara, G., & Simion, F. (2019). A mental number line in human newborns. *Developmental Science*, 22 , e12801.

Sarnecka, B. W., Kamenskaya, V. G., Yamana, Y., Ogura, T., & Yudovina, Y. B. (2007). From grammatical number to exact numbers: Early meanings of ‘one’,‘two’, and ‘three’in English, Russian, and Japanese. *Cognitive Psychology*, 55, 136-168.
