# Supplementary material for: Recency and rarity effects in disambiguating the focus of utterance: A developmental study
Source: PLoS One. 2025 Feb 12;20(2):e0317433. doi: 10.1371/journal.pone.0317433 (PMC11819549; doi:10.1371/journal.pone.0317433)
Supplement: S2 File — (PDF) [file pone.0317433.s002.pdf]

2. Details of the Procedure

In addition to recording responses, participants were also recorded with a video camera. Images displayed on the monitor, video footage of the participants, and images shown on the touch screen were synthesized (VR-4HD, Roland) and recorded (HSV326, TreasLin). The experimenter observed the study's progress via the mixer device and controlled the task online, outside of the participant' visual field. The experimenter provided no feedback, except for a verbal cue indicating when to move on to the next trial.
