# Supplementary material for: Recency and rarity effects in disambiguating the focus of utterance: A developmental study
Source: PLoS One. 2025 Feb 12;20(2):e0317433. doi: 10.1371/journal.pone.0317433 (PMC11819549; doi:10.1371/journal.pone.0317433)
Supplement: S4 File — (PDF) [file pone.0317433.s004.pdf]

4. Calculating the probability of each choice

Participants were allowed to choose any number of targets, with no restrictions on the number chosen. If there are 9 targets and participants can choose any number, including zero (i.e., not choosing any targets at all), then each individual target has two states: chosen or not chosen Therefore, there are $2^9 = 512$ possible combinations of choices. This number includes the possibility that no targets were chosen. However, our participants were required to choose at least one target. So in this case, the number of possible choices is 511.
