# Supplementary material for: Recency and rarity effects in disambiguating the focus of utterance: A developmental study
Source: PLoS One. 2025 Feb 12;20(2):e0317433. doi: 10.1371/journal.pone.0317433 (PMC11819549; doi:10.1371/journal.pone.0317433)
Supplement: S5 File — (PDF) [file pone.0317433.s005.pdf]

5. The translation of the introduction video

Practice session

Quote: Many animals live here. Let’s look.

Four dogs appear.

Quote: what happens next?

Four cats appear.

Task session

Quote: Many monsters live here. Let’s look.

Nine black monsters appear.

Quote: what happens next?

Nine red monsters appear.
