# Supplementary material for: Recency and rarity effects in disambiguating the focus of utterance: A developmental study
Source: PLoS One. 2025 Feb 12;20(2):e0317433. doi: 10.1371/journal.pone.0317433 (PMC11819549; doi:10.1371/journal.pone.0317433)
Supplement: S6 File — (PDF) [file pone.0317433.s006.pdf]

6. The details for other strategies

The participants' responses were categorized based on the assumption that participants relied on a strategy to make decisions. It is also possible that the participants remembered the sequence incorrectly or chose options without any specific strategy, so the following categorization should remain speculative. Due to small sample size of each strategy, further statistical analysis between the categories was not performed.
