# Supplementary material for: Recency and rarity effects in disambiguating the focus of utterance: A developmental study
Source: PLoS One. 2025 Feb 12;20(2):e0317433. doi: 10.1371/journal.pone.0317433 (PMC11819549; doi:10.1371/journal.pone.0317433)
Supplement: S1 Table — (DOCX) [file pone.0317433.s007.docx]

| Table S1 The sequence of the practice session | | | | |
| --- | --- | --- | --- | --- |
| Inserted position | 1 | 2 | 3 | 4 |
| #4 | Dog | Dog | Dog | Cat |
| #4 | Dog | Dog | Cat | Cat |
| #4 | Dog | Cat | Cat | Dog |
| #4 | Cat | Dog | Dog | Cat |
| #4 | Cat | Dog | Cat | Dog |
| #4 | Cat | Cat | Cat | Cat |
| #2 | Dog | Cat | Cat | Dog |
| #2 | Cat | Dog | Dog | Cat |
| #2 | Cat | Dog | Cat | Dog |
| #2 | Cat | Cat | Cat | Cat |
