# Supplementary material for: Recency and rarity effects in disambiguating the focus of utterance: A developmental study
Source: PLoS One. 2025 Feb 12;20(2):e0317433. doi: 10.1371/journal.pone.0317433 (PMC11819549; doi:10.1371/journal.pone.0317433)
Supplement: S3 Table — (DOCX) [file pone.0317433.s009.docx]

| Table S3 The categorization of other selection strategies in adults in the Double-Rare-Events Condition. | | | | | | |
| --- | --- | --- | --- | --- | --- | --- |
| 1^st^ Rare event | 2^nd^ Rare event | All events (#-9 - #-1) | Frequent events | Recent chunk after a rare event | Rare and recent event | Unspecified strategies |
| #-6 | #-3 | 6 | 1 | 1 | 0 | 0 |
|  | #-2 | 5 | 1 | -(recent event) | 2 | 2 |
|  | #-1 | 2 | 0 | -(recent / rare event) | -(recent / rare event) | 1 |
| #-5 | #-3 | 8 | 2 | 0 | 0 | 5 |
|  | #-2 | 4 | 1 | -(recent event) | 0 | 0 |
|  | #-1 | 2 | 0 | -(recent / rare event) | -(recent / rare event) | 1 |
| #-4 | #-3 | 4 | 2 | 0 | 0 | 2 |
|  | #-2 | 4 | 1 | -(recent event) | 0 | 0 |
|  | #-1 | 3 | 0 | -(recent / rare event) | -(recent / rare event) | 0 |
