# Supplementary material for: Recency and rarity effects in disambiguating the focus of utterance: A developmental study
Source: PLoS One. 2025 Feb 12;20(2):e0317433. doi: 10.1371/journal.pone.0317433 (PMC11819549; doi:10.1371/journal.pone.0317433)
Supplement: S4 Table — (DOCX) [file pone.0317433.s010.docx]

| Table S4 The categorization of other selection strategies in adults in the UI Double-Rare-Events Condition. | | | | | | |
| --- | --- | --- | --- | --- | --- | --- |
| Rare event after the utterance | Rare event before the utterance | All events (#-6 - #-1) | Frequent events (#-6 - #-1) | Recent chunk after a rare event (#-6 - #-1) | Rare and recent event (#-6 - #-1) | Unspecified strategies |
| #+1 | #-3 | 1 | 0 | 0 | 1 | 7 |
|  | #-2 | 0 | 0 | -(recent event) | 2 | 6 |
|  | #-1 | 0 | 0 | 0 | -(recent / rare event) | 4 |
| #+2 | #-3 | 0 | 0 | 1 | 0 | 4 |
|  | #-2 | 0 | 0 | -(recent event) | 2 | 5 |
|  | #-1 | 0 | 0 | 0 | -(recent / rare event) | 7 |
| #+3 | #-3 | 0 | 0 | 2 | 0 | 9 |
|  | #-2 | 0 | 0 | -(recent event) | 2 | 6 |
|  | #-1 | 0 | 0 | 0 | -(recent / rare event) | 5 |
