# Supplementary material for: Recency and rarity effects in disambiguating the focus of utterance: A developmental study
Source: PLoS One. 2025 Feb 12;20(2):e0317433. doi: 10.1371/journal.pone.0317433 (PMC11819549; doi:10.1371/journal.pone.0317433)
Supplement: S5 Table — (DOCX) [file pone.0317433.s011.docx]

| Table S5 The categorization of other selection strategies in children in the Single-Rare-Event Condition. | | | | | |
| --- | --- | --- | --- | --- | --- |
| Rare event | All events (#-9 - #-1) | Frequent events | Recent chunk after a rare event | Rare and recent event | Unspecified strategies |
| #-6 | 1 | 2 | 0 | 0 | 2 |
| #-5 | 2 | 0 | 0 | 0 | 2 |
| #-4 | 1 | 1 | 0 | 1 | 5 |
| #-3 | 0 | 1 | 0 | 0 | 0 |
| #-2 | 1 | 2 | -(recent event) | 0 | 1 |
| #-1 | 2 | 2 | 0 | -(recent / rare event) | 2 |
