# Supplementary material for: Recency and rarity effects in disambiguating the focus of utterance: A developmental study
Source: PLoS One. 2025 Feb 12;20(2):e0317433. doi: 10.1371/journal.pone.0317433 (PMC11819549; doi:10.1371/journal.pone.0317433)
Supplement: S1 Fig — Each animal appeared with an accompanying sound indicating cat or dog. Each animal wore a different colored ribbon so that they could easily be differentiated. (DOCX) [file pone.0317433.s014.docx]

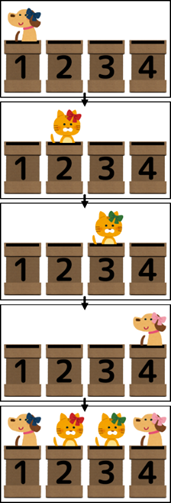
Figure S1 An example of a practice session. Each animal appeared with an accompanying sound indicating cat or dog. Each animal wore a different colored ribbon so that they could easily be differentiated.
