# Supplementary material for: Recency and rarity effects in disambiguating the focus of utterance: A developmental study
Source: PLoS One. 2025 Feb 12;20(2):e0317433. doi: 10.1371/journal.pone.0317433 (PMC11819549; doi:10.1371/journal.pone.0317433)
Supplement: S2 Fig — (DOCX) [file pone.0317433.s015.docx]

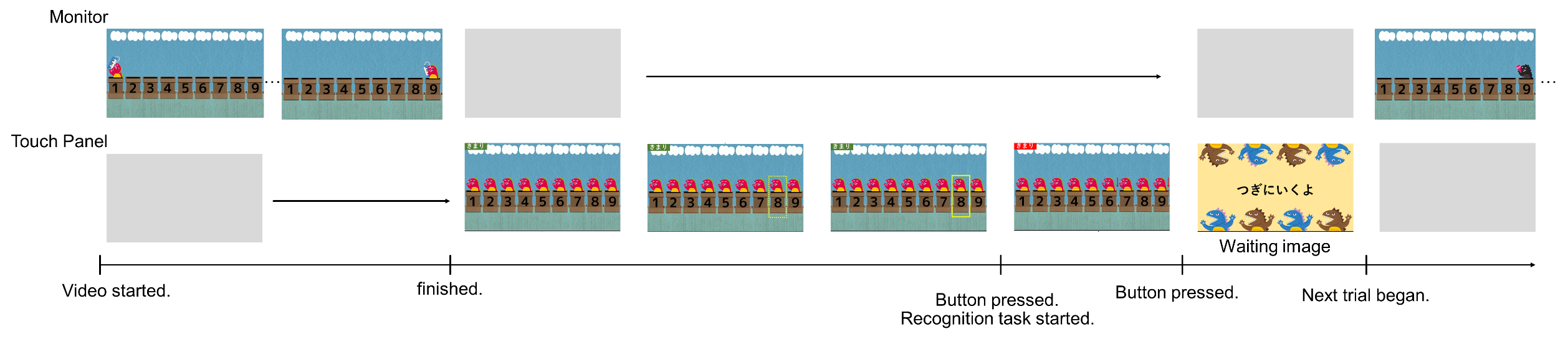
Figure S2 Schematic representation of the image on the monitor and touch screen. Note that the timeline is not to scale. The upper and lower lines represent the images on the monitor and on the touch screen, respectively. The touch screen did not present any images while a video was playing on the monitor. At the end of video stimulus, a visual image appeared on the touch screen. This methodology ensured that participants shifted their attention to the touch panel only after the video clip ended, and kept their attention on the video clip. If participants had been allowed to make a choice at any time, they might have responded right after the utterance, but before the video clip had finished. Maintaining attention throughout the video clip was crucial for the UI Double-Rare-Events Condition. Specifically, for the UI Double-Rare-Events Condition, the current study aimed to explore the participants' responses based on the observation of the entire sequence. A dashed line yellow rectangle was drawn by dragging the finger. Once a participant lifted his/her finger off the screen, the rectangle turned into solid line and was fixed into place. When participants touched the screen, the rectangle drawn was deleted.
