# Supplementary material for: Recency and rarity effects in disambiguating the focus of utterance: A developmental study
Source: PLoS One. 2025 Feb 12;20(2):e0317433. doi: 10.1371/journal.pone.0317433 (PMC11819549; doi:10.1371/journal.pone.0317433)
Supplement: S3 Fig — Distribution of response in (a) in Single-Rare-Event Condition, (b)Double-Rare-Events Condition and (c) UI Double-Rare-Events Condition, respectively. (DOCX) [file pone.0317433.s016.docx]

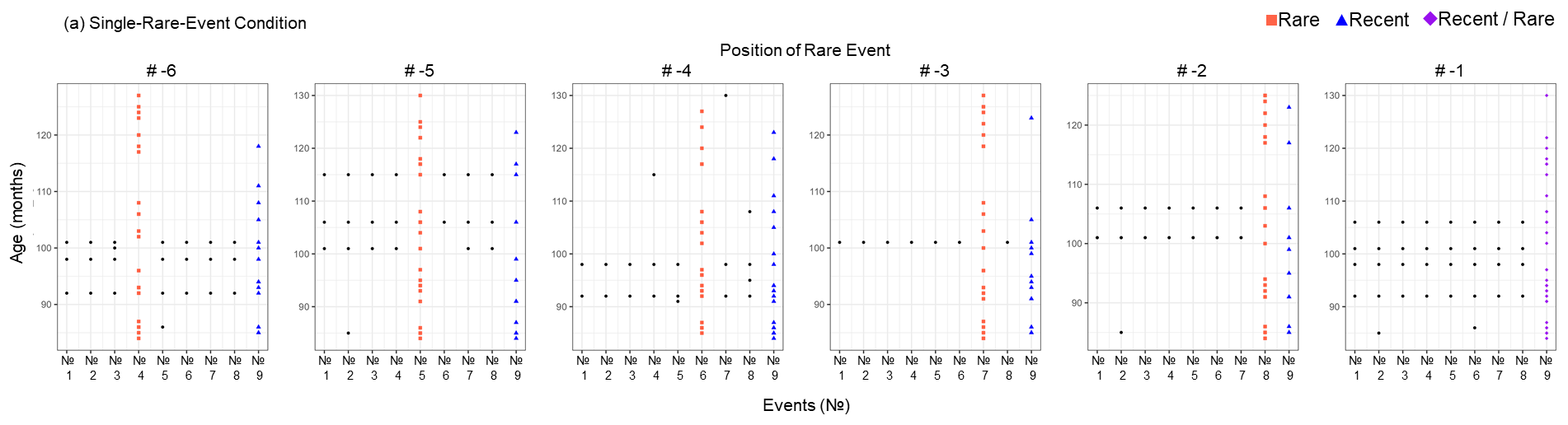


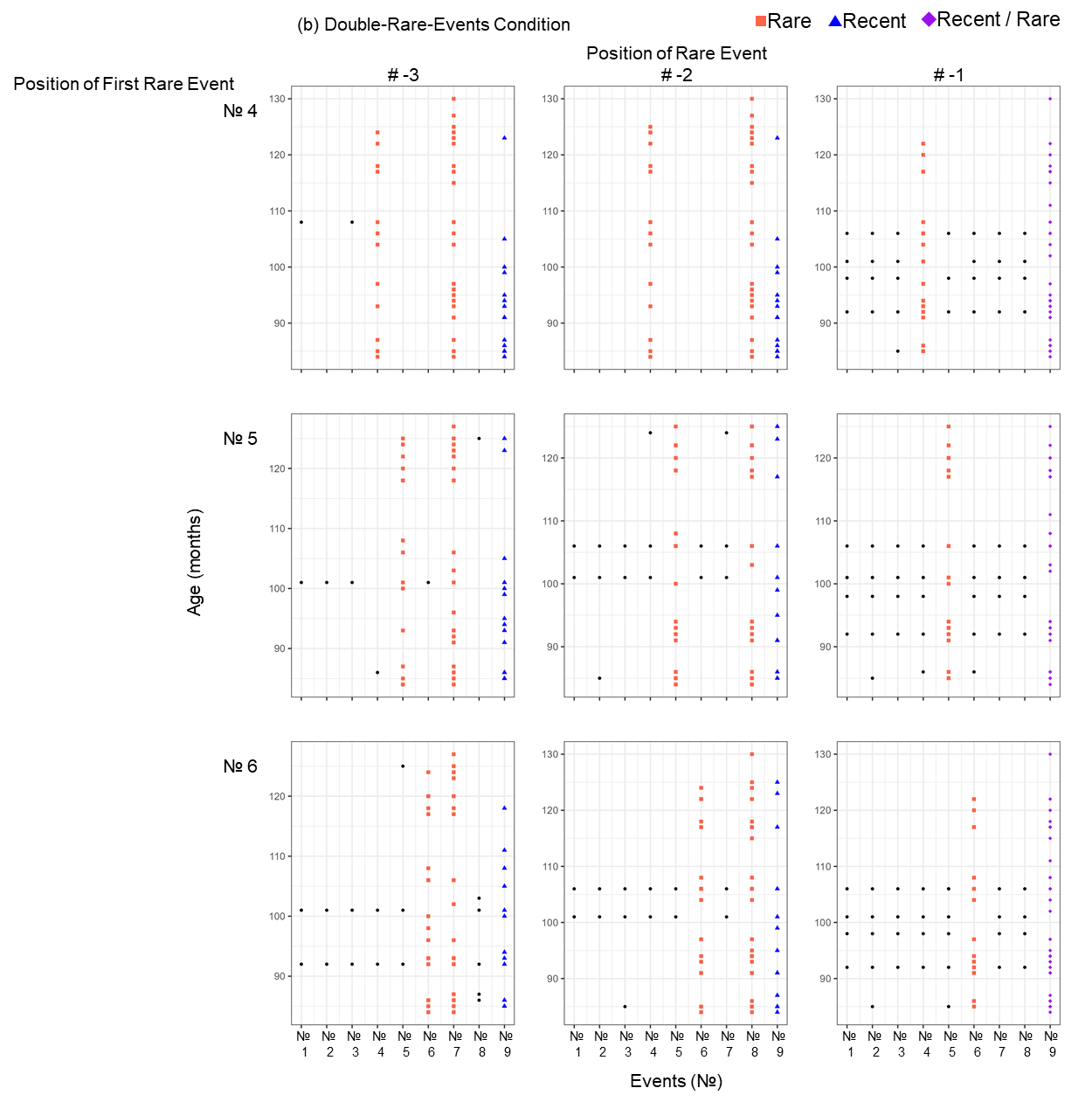


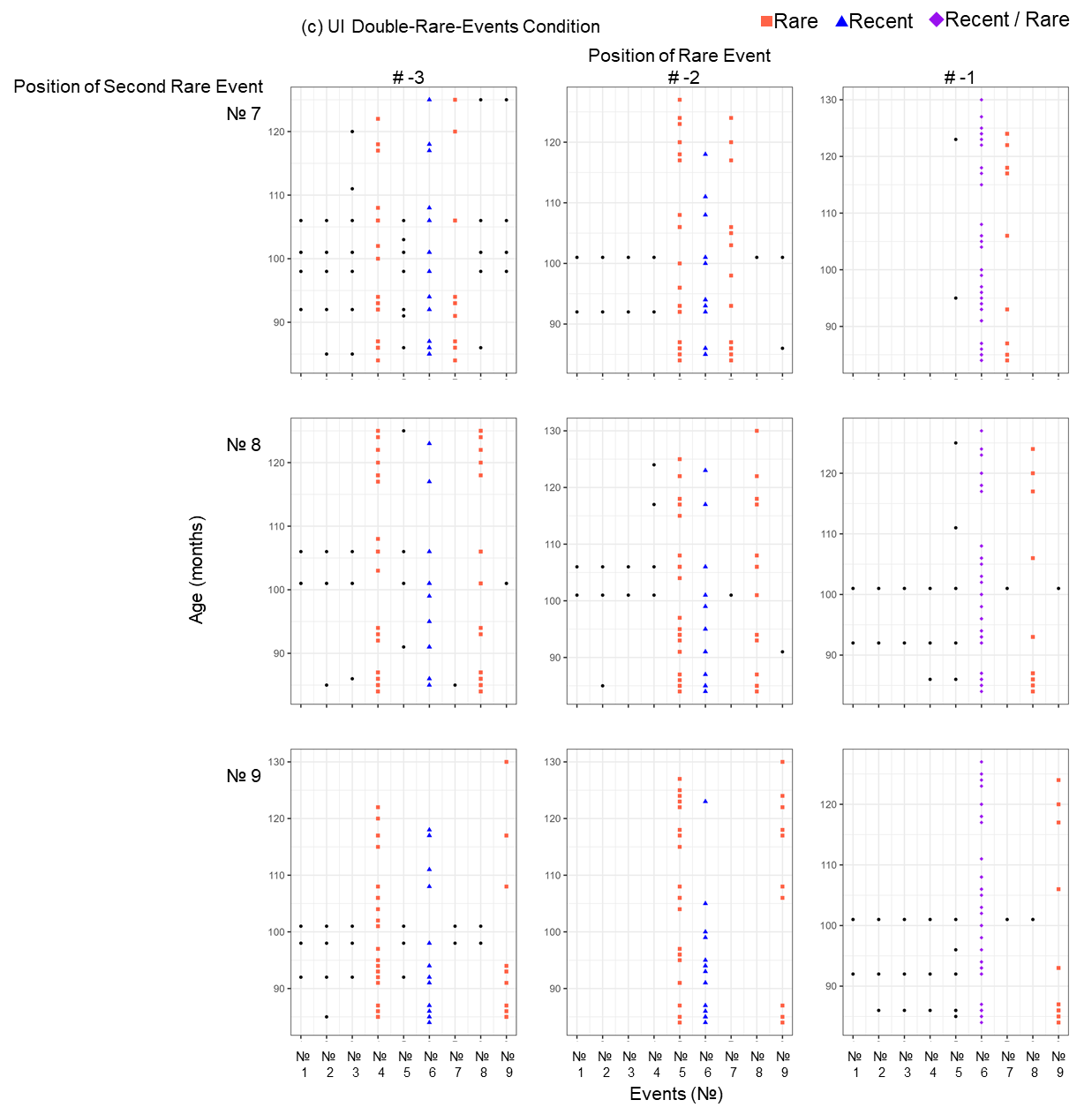


Figure S3. Distribution of response in (a) in Single-Rare-Event Condition, (b)Double-Rare-Events Condition and (c) UI Double-Rare-Events Condition, respectively. Each dot represents the event selected by a particular participant. X-axis represents each position of an event. Y-axis represents participants’ age in months. Red squares represent rare events, blue triangles represent recent events and purple diamonds represent rare / recent events.
