# Supplementary material for: Recency and rarity effects in disambiguating the focus of utterance: A developmental study
Source: PLoS One. 2025 Feb 12;20(2):e0317433. doi: 10.1371/journal.pone.0317433 (PMC11819549; doi:10.1371/journal.pone.0317433)
Supplement: S4 Fig — (DOCX) [file pone.0317433.s017.docx]

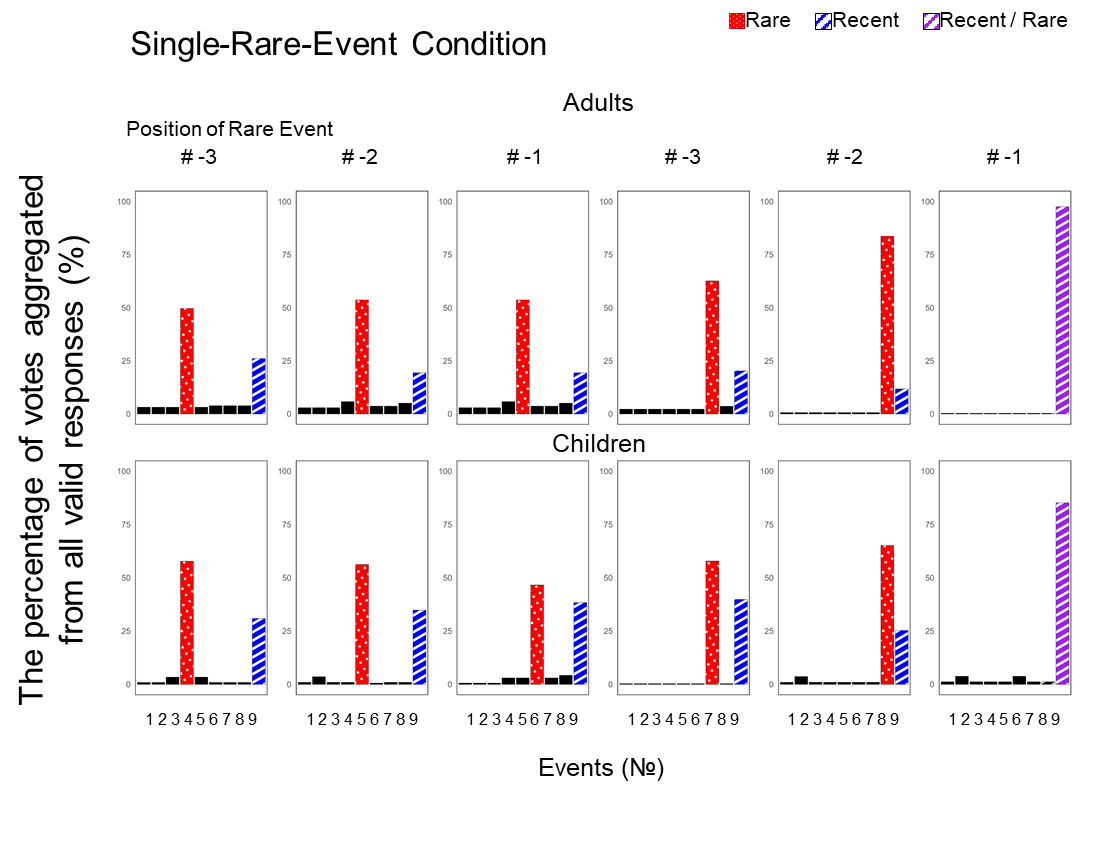
Figure S4 The distributions of responses for each option in the Single-Rare-Event Condition. It was assumed that each participant had one vote, which they could assign equally to any target. Thus, if a participant selected only #7, #7 received one full vote. If a participant selected #4, #7, and #9, each received 1/3 of a full vote, or 0.33. Results showed that few responses were made for targets other than rare or recent events (black-stiped stimuli).
