# Supplementary figures and images for: Recency and rarity effects in disambiguating the focus of utterance: A developmental study
Source: PLoS One. 2025 Feb 12;20(2):e0317433. doi: 10.1371/journal.pone.0317433 (PMC11819549; doi:10.1371/journal.pone.0317433)

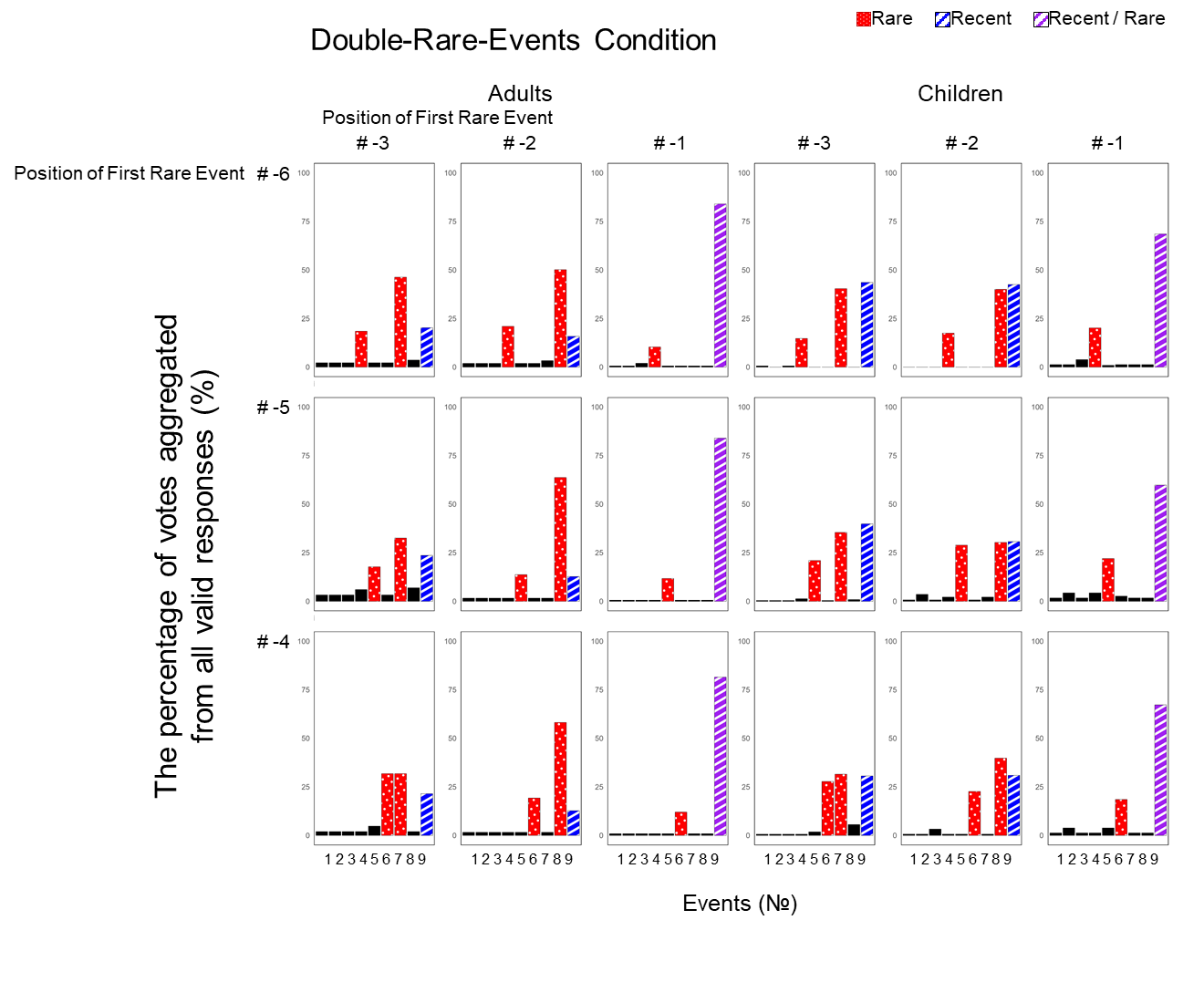
Figure S5 The distributions of responses on each option in the UI Double-Rare-Events Condition.

Supplement: S5 Fig — (DOCX) [file pone.0317433.s018.docx]

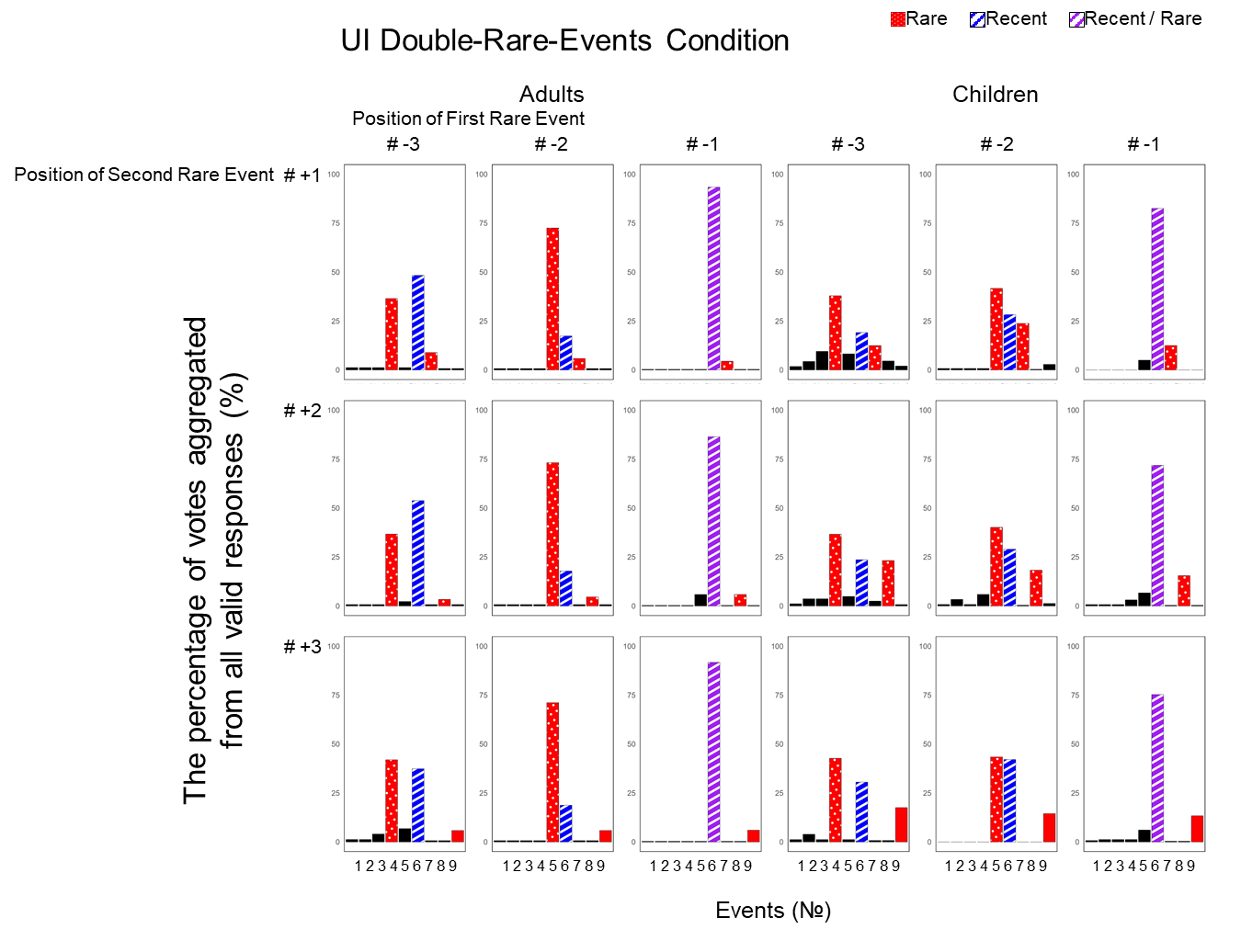
Figure S6. The distributions of responses on each option in the UI Double-Rare-Events Condition.

Supplement: S6 Fig — (DOCX) [file pone.0317433.s019.docx]
