# Supplementary material for: Recency and rarity effects in disambiguating the focus of utterance: A developmental study
Source: PLoS One. 2025 Feb 12;20(2):e0317433. doi: 10.1371/journal.pone.0317433 (PMC11819549; doi:10.1371/journal.pone.0317433)
Supplement: Attachment — Submitted filename: response_letter_final1118.docx [file pone.0317433.s020.docx]

**Reviewer #1:**


**Comment1-1:** I think Coding scheme (categorization of the participants’ responses) must be stated in detail. I understand that the participants put “rectangles” according to their interpretation of the communicator. This is a nice way to collect the participants’ interpretations because the participants can express their interpretation with more freedom than forced choice method. However, the categorization method sounds ambiguous.
Rectangles participants indicated may sometimes include more than one spot, such as one “rare” spot and one or two preceding spots (this may mean that “a change” occurred, from rare to not rare). How did you code such performance? I think it is necessary to describe the coding scheme completely in the text.

**Reply1-1:** The majority of participants’ response converged on a rare or a recent event. To prove this, we inserted new figures in supplementary materials (Figure S4 and Lines 324-329; Figure S5 and Lines 371- 372; Figure S6 and Lines 417 - 418) which shows the distribution of responses of each option. Specifically, in the Double-Rare-Events Condition, we believe that we should treat selecting only a latter rare event and selecting both rare events differently. It is because the aim is to explore the interaction of recency and rarity (Lines 374 - 376). In the UI Double-Rare-Events Condition, the question here is whether participants omit a rare event after the utterance from the candidate of referent (Lines 420 - 422). Thus, we treated selecting both rare events and selecting a rare event before utterance as different categories. We believe that our categorization scheme is an appropriate manner.

Tables S2 – S7 in supplementary materials shows that responses which cannot be categorized by any patterns. Some participants selected all options or against our prediction selected frequent events. We had not considered the possibility you suggested. We probed again responses categorized by unspecified strategies. However, the numbers of each response patters categorized by unspecified are too small. Additionally, these patters can reflect the possibility that participants remembered wrong sequences. Therefore, further exploration for these patterns is difficult. We inserted this explanation to reflect your comment (Page 11 in supplementary materials). We hope this satisfies your concerns.

**Lines 324-329 in Results | Single-Rare-Event Condition with Adult participants**

*“Visualizing the distribution of responses confirms that the majority of responses converged on rare or recent events (Figure S4 for details). It seems reasonable that participants were largely categorized into either those who selected a rare event or those who selected a recent event (rare-event timing #-1). Those who used other strategies such as selecting all events equally were categorized as others (Table S2 – S7 for other strategies observed in the current study).”*

**Lines 371 - 372 in Results | Double-Rare-Events Condition with Adult participants**

*“The participants responses converged on selecting one or two rare events or selecting a recent event (Figure S5 for details).”*

**Lines 374 – 376 in** **Results | Double-Rare-Events Condition with Adult participants**

*“Selecting the 2nd rare event and selecting both rare events were treated as separate categories because the question here is to examine the interaction of recency and rarity”*

**Lines 417 – 418 in Results| UI Double-Rare-Events Condition with Adult**

*“The participants response converged on choosing one or more rare events or choosing a recent event (Figure S6 for details).”*

**Lines 420 – 422 in** **Results| UI Double-Rare-Events Condition with Adult participants**

*“The question here was whether participants omit a rare event after the utterance as a candidate for a referent. Thus, selecting both rare events and selecting only a rare event before the utterance were treated differently.”*

**Page 11 in Supplementary material**

*“The participants' responses were categorized based on the assumption that participants relied on a strategy to make decisions. It is also possible that the participants remembered the sequence incorrectly or chose options without any specific strategy, so the following categorization should remain speculative.”*

**Comment1-2:** The results indicate that children treated recency and rarity cues separately, while adults treated recency as a continuous factor and integrated the gradient of recency with rarity. I’m not quite convinced about the authors’ statement. How do you know about being “separate” and being “continuous”? You seem to state it based on the AIC results, but still not very clear to me. More explanation is needed.

**Reply1-2:** Sorry for the lack of clarity. We were also confused and used the word of 'separate,' but your comments made us realize that it is unclear whether children separated or integrated rarity and recency only when recency peaked. Selecting a rare event when one occurred at #9 means choosing both a recent event and a rare event simultaneously. Thus, there are three possibilities: participants selected it as a rare event, as a recent event, or the interaction of recency and rarity mutually strengthened each other.

In both adults and children, the number of participants who selected a rare/recent event when a rare event occurred at #-1 is close to the sum of the participants who selected a rare or recent event when a rare event occurred at either #-3 or #-2. Thus, the highest number in #-1 might reflect the sum of participants who selected either a recent or rare event. However, adults showed linear increase between #-3 and #-2. This suggests adult treated recency as a continuous factor when integrating with rarity (Lines 584 - 591).

Children showed a nearly identical frequency of selecting a rare event between #-3 and #-2, with a noticeable increase only in #-1. This increase in #-1 might reflect the sum of participants who selected either a recent or rare event. If this possibility were dominant, they separated recency and rarity. However, another possibility is that they integrated recency and rarity only when recency peaked. Initially, we considered only the former possibility and thus argued “children treated recency and rarity separately”, but we have now included both possibilities in the current version of the manuscript (Lines 344 – 347, Lines 587 – 590 and Lines 631 - 632).

Children showed a nearly identical frequency of selecting a rare event between #-3 and #-2, with a noticeable increase only in #-1. This increase in #-1 might reflect the sum of participants who selected either a recent or rare event. If this possibility were dominant, they separated recency and rarity. However, another possibility is that they integrated recency and rarity only when recency peaked. Initially, we considered only the former possibility and thus argued “children treated recency and rarity separately”, but we have now included both possibilities in the current version of the manuscript (Lines 345 – 348, Lines 584 – 591 and Lines 632 - 633).

Except for the Double-Rare-Events Condition in adults, the number of times a rare event was selected when a rare event occurred at #-1 is close to the sum of the selections of either rare or recent events when a rare event occurred at #-2 and #-3 (Lines 582- 584; Lines 628 - 633). We assumed that the unique pattern in the Double-Rare-Events Condition is due to the stronger effect of rare events, leading some participants to choose both rare events (Lines 606-610). We now modified the sentence to reflect your comments.

**Lines 345 – 348 in** **Results | Single-Rare-Event Condition with Adult participants**

*“At this point, it was unclear whether participants used the interaction of rarity and recency. However, it was unclear whether they used these two factors in a separate manner or integrated them only when recency and rarity overlapped."*

**Lines 582 – 584 in Implications from comparing adults and children**

*“The number of participants who selected a rare/recent event when a rare event occurred at #-1 is close to the sum of the participants who selected a rare or recent event when a rare event occurred at either #-3 or #-2.”*

**Lines 584 – 591 in Implications from comparing adults and children**

*“the fact that the highest percentage of participants selected a rare/recent event at #-1 might indicate that participants did not integrate recency and rarity and treated them separately. However, adults showed a gradual increase between #-3 and #-2, suggesting that they treated recency as a continuous factor when integrating with rarity. However, a limitation of our study is that it remains unclear whether children separated recency and rarity as categorical / stable factors, or whether they integrated them only when recency peaked.”*

**Lines 606 – 610 in Implications from comparing adults and children**

*“The number of adults who selected a rare/recent event when a rare event occurred at #-1 is lower than the total number of participants who selected a rare or recent event when a rare event occurred at either #-3 or #-2. This could be attributed to the fact that presenting two rare events had a stronger impact, leading some adults to choose both rare events.”*

**Lines 628 – 633 in Implications from comparing adults and children**

*“Again, the number of participants who selected a rare event when a rare event occurred at #-1 is close to the sum of the participants who selected a rare or recent event when a rare event occurred at either #-3 or #-2. Adults showed a linear increase as a function of position of the rare event. Thus, adults integrated rarity and recency.”*

**Lines 632 – 633 in** **Implications from comparing adults and children**

*“Children again treated recency either as categorical and separate or integrated them only when recency peaked.”*

Trend analysis tested two hypotheses generated by our visual inspection. (1) Linear model assumes linear increase of selecting a rare event as a function of the position of a rare event. Thus, we replaced the position of a rare events #-3, #-2 and #-1 into -1, 0, 1, respectively. (2) Step model assumes a bounce of selecting a rare event when a rare event happened at #-1. Thus, we replaced the position of a rare events #-3, #-2 and #-1 into -0.5, -0.5, 1, respectively. These coefficients mean that #-3 and #-2 are the same value and only #-1 is higher than others. Lower AIC indicates better fitting of a model. Results showed that adults fitted to liner model better while children fitted to step model better along our hypothesis. To help readers understand this concept better, we now inserted detailed explanation of trend analysis (Lines 557 - 562) and modified the figure (see Figure 4). We hope this modification is your satisfactory.

**Lines 557 – 562 in Implications from comparing adults and children**

*“When a direction is clearly hypothesized, it can be expressed as a contrast [28]. This is the case for the current study because it also explored hypotheses generated through visual inspection. If a hypothesis predicts a particular pattern, this pattern can be represented numerically by assigning specific values to each condition. These assigned values, or contrast weights, capture the hypothesized differences in the means across experimental conditions.”*

**Comments 1-3:** UII Double-Rare-Events
What does UII stand for? Could you tell me what kind of abbreviation (if it is an abbreviation) it is?

**Reply1-3:** We apologize for the confusion. We forgot to explain the abbreviation, and have now added the explanation. In response to a native speaker’s advice, UII Double-Rare-Events Condition was replaced into UI Double-Rare-Events Condition (*Utterance In-between Double-Rare-Events Condition*).

**Lines 256 - 257**

*“Utterance In-between Double-Rare-Events Condition (hereafter UI Double-Rare-Events Condition)”*

**Comments 1-4:** Sometimes one sentence is too long and difficult to read. Please state the content more
compact and concisely. For example,

The percentage of choosing both
404 1st and 2nd rare events (which is equivalent to rare events before and after the
405 utterance in the UII Double-Rare-Events Condition) decreased in the UII Double-Rare-
406 Events Condition compared to the Double-Rare-Events Condition (95 of 324 (29.32%)
407 in Double-Rare-Events Condition vs. 31 of 318 (9.75%) in UII Double-Rare-Events
Condition, χ2(1) 408 = 40.55, p < .001, Odds ratio = 0.26), suggesting that adults recognized
409 the difference between these two conditions and employed distinct strategies for each.

**Reply1-4:** We now corrected the sentence you pointed out (Lines 425 – 430). We again read our manuscript thoroughly, we shortened several sentences as follows (Lines 598 – 601; Lines 645 – 647; Lines 776 - 779).

**Lines 425 – 430 in** **Results| UI Double-Rare-Events Condition with Adult participants**

*“The percentage of choosing both 1st and 2nd rare events (which is equivalent to rare events before and after the utterance in the UI Double-Rare-Events Condition) decreased in the UI Double-Rare-Events Condition compared to the Double-Rare-Events Condition (95 of 324 (29.32%) in Double-Rare-Events Condition vs. 31 of 318 (9.75%) in UI Double-Rare-Events Condition, χ2(1) = 40.55, p < .001, Odds ratio = 0.26).”*

**Lines 598 – 601 in Implications from comparing adults and children**

*“Children more frequently selected a recent event than adults did. This indicated that children’s interpretation of the utterance was inclined to recency when the relative frequency of rare events increased to 2 / 9 from 1 / 9 (in the Single-Rare-Event Condition) and the effect of rarity was thus weakened.”*

**Lines 645 – 647 in Discussion**

*“Across the three conditions, adults showed a linear increase in selecting a rare event as it approached the utterance. In contrast, children showed a bounce in selecting a rare event when a rare event occurred right before the utterance.”*

**Lines 776 – 779 in General Discussion**

*“Specifically, adults might perceive "Did you see that?" as a polysemous utterance that varies depending on the communicator’s interest, focus, perspective, experience or belief. In contrast , children interpret the same phrase more semantically, in other words, in a more literal manner.”*

**Comments 1-5:** In the Supplementary material “… that two read arrows were overlaid” -> should be “… that two red arrows were overlaid”

**Reply1-5:** Thank you for pointing out. We now corrected the sentence you pointed out.

**Comments 1-6:** 446the percentage of people 　　-> the percentage of children　
In the text, sometimes “children” and “people” seem to be mixed. “Children” sounds better.

**Reply1-6:** We reflect your comments by replacing people into “children” or participants for adults. In addition to your pointed here, we made this reflection through entire manuscript (Lines 465-469; Lines 477 - 482).

**Lines 465 – 469 in Results | Single-Rare-Event Condition with Child participants**

“The same analysis as used……, which posits that the percentage of children that chose rare events is not different from those that chose recent events. …… *The results showed that the percentage of children that chose rare events significantly higher from that of recent events……”*

**Lines 477 – 482 in Results | Double-Rare-Events Condition with Child participants**

*“We tested the null hypothesis that the percentage of children……The results showed that the percentage children that chose…… the percentage children that chose a recent event…….”*

**Comments 1-7:** Figure 2　　The vertical axis represents how far the 4 rare event from the utterance.
“Vertical “ axis -> should be “horizontal” axis, I think.

**Reply1-7:** Thank you for finding this miss. The horizontal axis represents how far the rare event from the utterance. We now corrected captions in Figures 2 and 3.

**Captions in Figure 2 and 3**

*”The horizontal axis represents how far the rare event from the utterance.”*

**Comments 1-8:** In the “Data of the figures, circumflex or hut　^-#1　is difficult to understand. What is the difference between -#1 and ^-#1 ? I thought that it means “excluding -#1 performance.” Is this correct? Anyway, please explain the meaning of ^-#1, and/or more understandable description of it in the figure is desirable.

**Reply1-8:** We agree with you in that “excluding -#1” captures the essence of here better. We now changed the circumflex of Figures 2 and 3 to reflect your comments.

**Comments 1-9:** In Figure ４　data points for adults and children look similar (with a slight color difference only)
**Reply1-9:** We now changed the color of lines and markers to reflect your comments. Please see Figure 4.

**Comments 1-10:** In the Double-rare condition, 　only -1,-2,-３ are described. How about -4, -5, -6? In my understanding, these distances might have existed according to the illustration of the stimuli.

**Reply1-10:** Sorry for the lack of clarity. The horizontal axis represents the position of the more recent event that occurred closer to the utterance (i.e., the second rare event). There are nine trials in total, consisting of a combination of three positions for the first rare event (-4, -5, and -6) and three positions for the second rare event (-1, -2, and -3). The focus here is on how the position of the second rare event affects disambiguation. Therefore, we aggregated the data within the second rare event. Please see the captions of Figure 2 and 3.

**Figures 2 and 3**

*“(b) The horizontal axis represents the position of a more recent rare event which occurred closer to the utterance.”*

**Reviewer #2:**

**Comments 2-1:**・In the Introduction, the use of terms such as "epistemic state" and "entropy" provides a theoretical framework for understanding the cognitive processes under investigation. However, these terms are scarcely referenced in the Discussion section. Instead, the Discussion primarily focuses on "common ground" and the concept of "we," which creates a disconnect between the Introduction and the Discussion. It would improve clarity if the Discussion were more closely aligned with the Introduction, integrating the theoretical terms initially introduced to maintain coherence throughout the argument.

**Reply2-1:** We must admit our discussion too focused on the concept of common ground and lacked the perspective assessed in Introduction. We now inserted a new paragraph which explains how our results can be interpreted from the concept of entropy, epistemic state, surprise and rarity. We hope this modification reflects your comments (Lines 715 - 728).

**Lines 715 – 728 in General discussion**

*“Whereas children have……In adults, the P300 amplitudes in response to oddball stimuli increased as the probability of standard/deviant stimuli increased; in other words, when oddball stimuli became rarer. Infants as young as 6 months have shown sensitivity to deviant stimuli [29], suggesting the attention to rare event in an epistemic state from early development to adulthood. The stimuli in the current study had the same structure as in the aforementioned oddball task (a monster who played a different action was an oddball). Participants formed an expectation that the same event would recur. When a rare event occurred, their expectation was violated, resulting in surprise. We developed our hypothesis based on the concept of entropy, surprise, memory, and causality. The results showed that our participants across both age groups integrated these factors by attributing their perception to others.”*

**Comments 2-2:** The studies cited in the previous studies focus on infancy and early childhood, yet this study targets children aged 7-10. It is essential to clarify why this age group was selected and to provide a theoretical justification for its significance in the context of development. Specifically, discussing how this age range might represent a transitional phase in the development of pragmatic skills related to referential disambiguation would strengthen the theoretical positioning of the study.

**Reply2-2:** The practical reason for selecting this age range is that seven may be the minimum age at which participants can effectively engage with our task (Lines 208–209). When targeting younger age groups, some procedural development would be necessary. This remains a task for future studies (Lines 840–842).

Children aged 7 to ten is still in the process of perceiving an established common ground with others. We now cited several studies which explored relatively older children’s perception of common ground. We hope this modification strengthen the theoretical rationale for the selection of this age range (Lines 210 - 221). We also believe that discussion about how children perceived shared experience in Introduction bridges the discrepancy between introduction and discussion you pointed out in **Comments 2-1**.

We discussed the reason why our children who are relatively old did not show similar pattern as adults. Thirteen-year-old children have not yet reached adult levels in the perceptual task which required to switch the task (Davidson et al., 2006). Considering the recency gradient and then integrating it with rarity requires greater cognitive flexibility then treating them as constant. This might explain why the children in our study did not show patterns different from adults (Lines 682 - 690).

**Lines 208 – 209 in Introduction**

*“*for practical reasons, selected participants were seven to ten years of age.*”*

**Lines 210 – 221 in Introduction**

*“Another reason why the current study chose children aged seven and older is that seven or eight years of age is a transitional phase in which children learn to perceive a common knowledge state with others. One study demonstrated that children aged six to seven perceived an shared knowledge with others only when they received minimal communicative cues [25]. Children aged six to eight develop the ability to recognize others' knowledge or ignorance, which may differ from their own. Between ages four and eight, the percentage of children who could correctly recognize others' knowledge states increased. Thus, at eight, they are still in the process of the development in perceiving shared experiences. One aspect of the current study explores how children disambiguate an utterance based on their perceived shared experience, which potentially leads to common ground. Because of these reasons, we tested children aged seven and older.”*

**Lines 682 – 690 in General Discussion**

*“**In the current study, older children did not show the same patterns as adults. Children in this age range are still in the process of understanding others' degree of knowledge [25]. However, in this study, the dominant cues available were their own salient cues, viewed from the communicator’s perspective. Research has shown that cognitive flexibility, especially in switching between tasks, has a longer developmental trajectory. Thirteen-year-old children have not yet reached adult levels [30]. Considering the recency gradient and then integrating it with rarity requires greater cognitive flexibility then treating both factors as categorical. This might explain why the children in our study did not show patterns different from adults.”*

**Lines 840- 842 in General Discussion**

*“Future studies across a wider cultural or linguistic background including younger age exploring interaction between cultural and developmental effects would provide a new perspective to this area of study.”*

**Comments 2-3:** Regarding the results, the use of the trend analysis as a method to assume a model is not sufficiently justified. A more explicit rationale for employing this method, including a discussion of why it is particularly suitable for the current dataset and the types of patterns it aims to uncover, would be beneficial.

**Reply2-3:** We replied this point which reviewer 1 also raised (please see also Comments 1-2). We now modified the figure (Figure 4) to help readers understand the concept of our analysis. We also now inserted a detailed explanation why we believe our analysis is valid (Lines 557 - 562).

**Comments 2-4:** Line 707: The description that "the communicator and participant had common ground" requires further clarification. While the description of "a shared visual experience at the time the utterance was made" is understandable, the current experiment appears to involve only a retrospective interpretation of the referent "that" in the utterance "Did you see that?" This raises doubts about whether such an interaction can truly be characterized as being based on common ground, especially given that no communicative behavior was observed between the experimenter and participant to explicitly establish this shared understanding during rare event.

**Reply2-4:** Thank you for your insightful comment. We must admit the description that "the communicator and participant had common ground" was and overstate. We agree with you in that the experimental setting made participants to perceive shared visual experience with the virtual communicator. We now revised the sentence as follows to reflect your comment (Lines 784 - 786). When discussion about common ground somewhere else (in **Comments 2-2**), We were again careful not to overstate (Lines 218 - 221).

**Lines 218 – 221 in Introduction**

*“One aspect of the current study explores how children disambiguate an utterance based on their perceived shared experience, which potentially leads to common ground.”*

**Lines 784 – 786 in General Discussion**

*“The setting of the current study assumes that the (virtual) communicator and participant shared the same visual experience at the time the utterance was made.”*

**Comments 2-5:** Furthermore, the discussion about "we" is intriguing, particularly the idea that a referential frame of "we" emerges when both the experimenter and participant are included in the events. However, it would be more effective to separate this point from the discussion of the disambiguation of "that" and the concept of "we-ness." Doing so would allow for a clearer examination of each concept's contribution to the overall findings.

**Reply2-5:** Thank you again for your insightful comments. To help readers understand our argument, we now modified the order of sentences (Lines 783 - 784). The topic sentence was put in the heading of a paragraph. The discussion of we-ness from the current experiment might be an overstate. So, to tell readers that the focus of our discussion here is the issues of translation for future cross-cultural or linguistic studies, we now inserted one sentence (Lines 821 - 823).

**Lines 783 – 784 in General Discussion**

*“The current study took the English translation, "Did you see that?" from the original Japanese phrase, "Ima-no-mita?"…….*

**Lines 821 – 823 in General Discussion**

*“This perspective should be important for future studies in languages where the translation of "Did you see that?" includes or does not include the subject, regardless of whether it does or does not.”*
