# Supplementary material for: Recency and rarity effects in disambiguating the focus of utterance: A developmental study
Source: PLoS One. 2025 Feb 12;20(2):e0317433. doi: 10.1371/journal.pone.0317433 (PMC11819549; doi:10.1371/journal.pone.0317433)
Supplement: Attachment — Submitted filename: Response_letter1218.docx [file pone.0317433.s021.docx]

**Comment 1-1:** 684 However, in this study, the dominantdominant cues available we

688 Considering the recency gradient and then integrating it with rarity requires
689 greater cognitive flexibility then treating both factors as categorical.
 There are two “then(s).” May be the following sounds better with “,”.

In the Supporting information
Figure S5 The distributions of responses on each option in the UDouble-Rare-Events Condition
Should be UI.

**Reply:** Thank you for pointing out our typos. We replaced then into than to reflect your comment. As for the second part, we believe “than” sounds better.

**Lines 687 - 688**

*“However, in this study, the dominant cues available were their own salient cues, viewed from the communicator’s perspective.”*

**Lines 691 - 692**

*“Considering the recency gradient while integrating it with rarity requires greater cognitive flexibility than treating both factors as categorical.”*

**Page 25 in supplementary information**

*“Figure S5 The distributions of responses on each option in the UI Double-Rare-Events Condition.”*

**Comment 1-2:**In the text

In adults, the P300 amplitudes in response to oddball stimuli increased as the
729 probability of standard/deviant stimuli increased; in other words, when oddball stimuli
730 became rarer. Infants as young as 6 months have shown sensitivity to deviant stimuli
731 [29], suggesting the attention to rare event in an epistemic state from early
732 development to adulthood. The stimuli in the current study had the same structure as in
45
the aforementioned oddball task (a monster who 733 played a different action was an
734 oddball).
->
This part is completely redundant. Please check the entire manuscript for redundancy.

**Reply:** You comments made me be aware that this paragraph repeated the same content. We edited this part to reflect your comments.

**Lines 720 - 735**

*“In adults, P300 amplitudes in response to oddball stimuli increased as their probability decreased. Sensitivity to deviant stimuli has been observed in infants as young as 6 months [29], suggesting that attention to rare events develops early and persists into adulthood. The stimuli in the current study mirrored the oddball task structure, with a monster performing a different action serving as the oddball. Participants formed an expectation that the same event would recur, and when a rare event occurred, their expectation was violated, resulting in surprise. We developed our hypothesis based on the concept of entropy, surprise, memory, and causality. The results showed that participants across both age groups integrated these factors by attributing their perception to others. Children have already begun to understand that an element they perceive to be salient in a conversational context is likely to be perceived as salient by others as well. This belief allows them to pinpoint what is relevant for them from the communicator’s perspective. In the current paradigm, participants and their communicative partner observed novel events together. Participants had a belief that they shared the same knowledge and epistemic state with the communicative partner and that the partner was also aware of this belief.”*

**Comments 1-3:** Please check the entire manuscript for redundancy.

**Reply:** We carefully reread our manuscript and realized that we frequently used multiple words in parallel with 'or' or 'and'. In most cases, however, the first word alone was sufficient to convey our ideas. Additionally, we often used unnecessary adjectives and adverb. Therefore, we omitted redundant words and edited some sentences in line with your comments. Additionally, we made several edits to address other redundant sentences.

We have already proceeded with the minor revisions. We acknowledge that some sentences still contain redundancy; however, we are concerned about altering the intended meaning. As a result, we made only minimal changes. We will take your advice into careful consideration for future work.

**The deleted parts are marked with lines.**

**Lines 47 - 48**

*“Since any social species ~~including humans~~ cannot exchange their perspectives as long as they have independent minds, they rely on shared communicative rules [2, 3].”*

**Lines 53 - 55**

*“Mary attributes “that” to the most salient event, despite numerous other possibilities like the clouds, stars, or moon.”*

**Lines 59 - 60**

*“This fact means that receivers do not always succeed in decoding the communicator’s focus ~~correctly~~.”*

**Lines 64 - 65**

*“Humans ~~clarify referential ambiguities~~ disambiguate information by discerning what information would a communicator perceive worthy from a receiver’s perspective.”*

**Lines 68 - 70**

*“As a result of this mutual process, the receiver attributes the communicator’s utterance to the relevant objects ~~or events~~ by … … [7].”*

**Lines 72 - 73**

*“They behave as if they were aware that the other individual ~~sees, hears,~~ perceives, processes or memorizes … ….”*

**Line 89**

*“Our focus here is how children calculate ~~and/or integrate~~ multiple factors.”*

**Lines 102 - 104**

*“Thus, their expectation is violated when a different event occurs [12]. ~~Humans perceive consistency and expect a familiar event to recur when a series of repeated events is identical.~~ When events occur infrequently, a surprising event stands out as ~~new or~~ unfamiliar.”*

**Lines 114 - 116**

*“This tendency increases over development [7, 15]. ~~Humans are sensitive to rarity in addition to recency.~~ The next question here is how children use other salient cues and whether these cues are outweighed by temporal proximity.”*

**Lines 131 - 133**

*“Another perspective supporting … … is the observation that animals tend to remember recent events more ~~strongly and~~ vividly [19].”*

**Lines 143 - 145**

*“The current study explored the development of pragmatic reasoning to identify … …what the communicator thinks is relevant for the receiver ~~when they share perceptual information~~.”*

**Lines 171 - 174**

*“Additionally, … …, it is hypothesized that when a rare event occurred at any point during a sequence of events, ~~and its timing varied,~~ the degree of recency of this rare event increased… ….”*

**Lines 291 - 293**

“The first three monsters performed … … a different event would be perceived as ~~infrequent and~~ rare.”

**Lines 298 - 299**

*“They pressed a decision button on the upper left to proceed to the memory task~~, which tested whether participants correctly remembered when the utterance was presented~~.”*

**Lines 538 - 540**

*“The percentage of those … … to explore whether adults and children had the same inclination toward recency~~, or whether they differed~~.”*

**Lines 653 - 655**

*“The first (Single-Rare-Event) condition showed that, ~~in the process of disambiguation,~~ adults and children alike used both rarity and recency as cues but depended more on rarity.”*

**Lines 773 - 774**

*“Children’s interpretation of the demonstrative “that” might be restricted to the ~~spatial or~~ temporal regulation of the literal “that”.”*

**Lines 822 - 823**

*“they might* *discern that the communicator intends to share surprising ~~or unexpected~~ events that the communicator and receiver recently experienced together.”*
