# Supplementary material for: Recency and rarity effects in disambiguating the focus of utterance: A developmental study
Source: PLoS One. 2025 Feb 12;20(2):e0317433. doi: 10.1371/journal.pone.0317433 (PMC11819549; doi:10.1371/journal.pone.0317433)
Supplement: Attachment — Submitted filename: PLOSONE1226_ResponseLetter.docx [file pone.0317433.s022.docx]

**Typographical corrections**

[2.26] answered -> identified
[3.34] utterance -> utterances
[3.36] Delete "the" before "reasoning"
[4.43] Delete "the" before "information"
[4.46] Add "information" after "what;" delete "of the information"
[4.49] Delete "the" before "referential assignment;" delete "a" before "pragmatic"
[4.50-4.51] Delete "into;" delete "the" before "communicative" replace "which" with "that"
[4.53] Add "is" before "passing"
[5.58-5.59] the what makes Peter refer -> what Peter is referring to
[5.62] Delete "The" at beginning of sentence; replace "the one" with "that"
[5.63] Replace "which" with "that"
[5.65] would a communicator -> a communicator would
[5.68] shoe -> shoes
[5.71] have a belief -> believe
[6.79] Delete "each"
[6.80] the typical hearing -> individuals with typical hearing
[6.84] no additional -> minimal
[7.105] Add "is" before "consequently"
[8.112] which -> that
[8.118] Add "that" after "states"
[12.183] perspectives -> perspective
[15.237] University's -> University; staffs -> staff
[20.316] by R core team -> in R (add citation for R core team); add "the" after "with"
[21.332] Add "was" before "significantly;" from -> than
[23.367] participants -> participants'
[24.377] Add "was" before "significantly;" from -> than
[24.382] Add "was" before "significantly;" from -> than
[26.436-26.437] Add "was" before "significantly;" from -> than
[28.451] less -> fewer
[29.462] Delete "as" before "used"
[33.526] Add "a" before "strategy"
[33.530] standing -> considering
[33.541] Add "the" after "with"
[34.548] Add "the" after "in"
[34.551] Delete "onward;" insert comma after "children"
[35.576] Add "of" after "in favor"
[38.615] Evens -> Events
[40.543] bounce -> sharp increase
[40.645] which -> that
[40.651] the -> a
[41.663] which -> that
[41.666] bounce -> sharp increase
[42.687] Delete "semantically"
[42.692] And -> Moreover,

**Reply:** We have carefully re-checked all the typographical corrections you pointed out and confirmed their accuracy. The corresponding sentences have been revised accordingly in response to your suggestions.
